# Differences in pharmaceutical consumption and expenses between immigrant and Spanish-born populations in Lleida, (Spain): A 6-months prospective observational study

**DOI:** 10.1186/1472-6963-8-35

**Published:** 2008-02-06

**Authors:** Montserrat Rue, Maria-Catalina Serna, Jorge Soler-Gonzalez, Anna Bosch, Maria-Cristina Ruiz-Magaz, Leonardo Galvan

**Affiliations:** 1 Biomedical Research Institut, Lleida (IRBLLEIDA), Spain; 2 University of Lleida, Lleida, Spain; 3 Regional Primary Care Management Office, Catalan Institute of Health, Lleida, Spain; 4 Rambla de Ferran Health Center, Catalan Institute of Health, Lleida, Spain; 5 Eixample Primary Care Health Center, Catalan Institute of Health, Lleida, Spain; 6 Catalan Health Department, Lleida, Spain

## Abstract

**Background:**

There are few studies comparing pharmaceutical costs and the use of medications between immigrants and the autochthonous population in Spain. The objective of this study is to evaluate whether there are differences in pharmaceutical consumption and expenses between immigrant and Spanish-born populations.

**Methods:**

Prospective observational study in 1,630 immigrants and 4,154 Spanish-born individuals visited by fifteen primary care physicians at five public Primary Care Clinics (PCC) during 2005 in the city of Lleida, Catalonia (Spain). Data on pharmaceutical consumption and expenses was obtained from a comprehensive computerized data-collection system. Multinomial regression models were used to estimate relative risks and confidence intervals of pharmaceutical expenditure, adjusting for age and sex.

**Results:**

The percentage of individuals that purchased medications during a six-month period was 53.7% in the immigrant group and 79.2% in the autochthonous group. Pharmaceutical expenses and consumption were lower in immigrants than in autochthonous patients in all age groups and both genders. The relative risks of being in the highest quartile of expenditure, for Spanish-born versus immigrants, were 6.9, 95% CI = (4.2, 11.5) in men and 5.3, 95% CI = (3.5, 8.0) in women, with the reference category being not having any pharmaceutical expenditure.

**Conclusion:**

Pharmaceutical expenses are much lower for immigrants with respect to autochthonous patients, both in the percentage of prescriptions filled at pharmacies and the number of containers of medication obtained, as well as the prices of the medications used. Future studies should explore which factors explain the observed differences in pharmaceutical expenses and if these disparities produce health inequalities.

## Background

Immigration in Spain has increased dramatically in the last decade. In 1996, immigrants represented 1.4% of the total Spanish population, and by 2006 had reached 10.8% [1].

In Spain, there is a National Health System (NHS), financed mainly by taxes, which provides universal and free health coverage including primary, specialized and hospital health care [2]. Primary care physicians represent the entry point to the health system. In Spain, immigrants may register in their municipality of residence to have access to health care, regardless of their legal status. However, a number of immigrants are not registered because they are unaware of the process, fear legal authorities' access to the database, or their municipal government rejects their registration [3]. Some immigrants, primarily of sub-Saharan origin, wait for weeks to register with the municipal authorities because they do not have a passport or other documentation to validate their identity. Other immigrants register very rapidly.

Pharmaceutical consumption may be related to the health care system of the country and the type of insurance of the user. Lundberg *et al *studied the effect of user charges on the use of prescription medicines [4]. They concluded that the young, those with poor health status, low education and low income are most likely to decrease consumption of prescription drugs when user charges increase. In Spain, user co-payments are restricted to pharmaceutics. The user pays 40% of the price of medication prescribed by NHS doctors (100% of private prescription drugs), with the exception of inpatients and 'exempt' groups (retired, handicapped, and people who have suffered occupational accidents) and drugs administered in hospitals, which are provided without cost to the patient. Drugs for chronic diseases are subject to 10% cost sharing when explicitly prescribed by NHS doctors to patients identified as chronically ill [5].

Due to the recent nature of the migratory phenomenon in our country, there are few studies of pharmaceutical costs and the use of medications in immigrant populations, and even fewer that compare their usage rates with those of the autochthonous population. A report by the BBVA-IEA foundation, directed by professor Guillem López-Casasnovas analyzed the impact of immigration and observed that, in Spain, immigrants consume less medication than expected [6]. Another study done in Almería examined prescriptions written at an emergency room to Muslims during Ramadan and observed that physicians adapted their prescribing practices to prevent non-compliance with treatment [7].

Gaskin et al, found that age and health status do not fully explain observed disparities in prescription drug use [8]. According to the authors, racial and ethnic inequalities may reflect the skepticism of patients with respect to medicine and health care in general, lower compliance with medical advice, communication problems between doctors and patients, and possibly differences in the prescribing habits of care-givers.

Nevertheless, according to community pharmacists, the pharmaceutical attention that immigrants receive does not differ from that provided to the autochthonous community, although they recognize that there are barriers that impede adequate intervention in these patients [9].

In Lleida (Catalonia, Spain), the urban immigrant population has grown from insignificant percentages some years ago to 17% of the resident population in December 2006 [10]. This increase in the immigrant population motivated us to evaluate whether there are differences in health care services provided to immigrants, with regard to pharmaceutical consumption and expenses, when compared to the autochthonous population.

## Methods

### Setting

Lleida is a city located in northeast Spain, in the autonomic region of Catalonia, with approximately 130,000 inhabitants, of which roughly 22,000 are immigrants. Its economy is based on agriculture and commerce. Immigrants living in the area are primarily economic immigrants, whose countries of origin have much lower incomes per capita than Spain. They are primarily men (approximately 60%) and younger, on average, than the Spanish-born population. Immigrants come mostly from Latin America, the Maghreb, sub-Saharan Africa and Eastern Europe.

### Study design

Prospective observational study in immigrant and Spanish-born populations.

### Study population

Patients visited by fifteen primary care physicians at five Primary Care Clinics (PCC), located in neighborhoods with the largest immigrant populations. These PCC belong to the Catalan Health Institute (ICS), the main public provider of health services in Catalonia.

The study was approved by the ethics committee of the Hospital Universitari Arnau de Vilanova (Lleida, Spain).

### Study period

From March 2005 to August 2005.

### Study sample and sample size considerations

Our sample size objective was to include approximately 385 individuals from each of the 4 largest groups of immigrants in the city (Latin America, Maghreb, sub-Saharan Africa and Eastern Europe), in order to estimate proportions by zone of origin with a sampling error of 0.05 or lower. Therefore, we planned to include approximately 1,600 immigrants. Since the age distributions of immigrants and Spanish-born individuals are different, we decided to select a random sample of 300 Spanish-born individuals from each of the 15 participating physicians. A sample of this size would allow us to assess associations between origin group and pharmaceutical consumption, adjusting by age and sex with multivariable models.

All immigrant patients visited by participating physicians during the study period were included in the study, a total of 1,630 immigrants. A sample of 4,500 Spanish-born patients was selected from patients that were not coded as immigrants. Of these individuals initially identified as Spanish-born, 346 were excluded after review by participating physicians because their country of origin was not coded or had errors, with the final number of autochthonous patients being 4,154. Thus, the total number of participants in the study was 5,784.

The study population was divided into groups by birth region: Spain, Maghreb, Eastern Europe, Latin America, Sub-Saharan Africa, other low-income countries (including Asian countries) and high-income countries [11,12]. High-income countries were excluded from the study because the sample was too small.

Native-born individuals were defined as individuals born in Spain and immigrants as individuals from low- and middle-income countries outside Spain.

### Variables and data collection system

In Catalonia, since October 2001, it has been obligatory for patients to use their health card to obtain medications in pharmacies. Pharmacies provide the Catalan Health Service with monthly computerized reports on each medication dispensed, a Personal Identification Code (PIC), and the prescription code, which are then linked with other information sources providing information on the doctor and health center that produced the prescription.

In Lleida, since 2001, there have been computerized applications to take advantage of this information, always ensuring the confidentiality of these data. Therefore, the source of information in our study comes from the prescription bills of pharmacies in Lleida, using a purpose built computerized data-collection system.

The variables used in our study are: date of birth, gender, number of containers of medication, and the price of the medication dispensed. In the analysis of medication dispensed, the ATC international classification (Anatomical Therapeutic Chemical Classification System), which was adopted by the WHO in 1996 and is official in Spain since 2003, was used. Taking the ATC classification as a baseline, eight large prescription groups were studied:

- Medications for pathological hypertension or cardiac insufficiency (therapeutic subgroups C02, C03, C07, C08, C09).

- Medications for diabetic conditions treated with insulin (subgroup A10A).

- Medication for diabetic conditions treated with oral anti-diabetics (subgroup A10B).

- Medications for hyperlipemia (therapeutic subgroup C10).

- Medications for asthma or COPD (therapeutic subgroup R03).

- Medication for mental illness (subgroups N05A, N05B, N05C y N06A).

- Medication for osteoarticular illness (subgroups M01, M02, M05, H05BA, G03XC).

- Medications for ulcerative conditions (subgroups A02A, A02B).

- Medications for infectious diseases (subgroup J01).

Each prescription group was analyzed during the study period to determine the number of patients treated, the amount of medication purchased, total expenditures for the group, and expenditure per patient, both for the immigrant population and the autochthonous population.

### Statistical Analysis

A descriptive analysis of pharmaceutical expenditures by age group, gender, immigrant/autochthonous group and region of origin was performed. A box-and-whisker plot, which summarizes continuous data using five  summary numbers (the lower quartile (Q1) minus 1.5 times the  inter-quartile range (Q3-Q1) or the smallest observation, the lower  quartile (Q1), the median, upper quartile (Q3), and Q3 plus 1.5 times the  inter-quartile range (Q3-Q1) or the largest observation), was used to  obtain a graphical comparison of pharmaceutical expenses.

Multinomial regression models were estimated using pharmaceutical expenditure as the dependent variable, which was classified into four categories based on expenditure quartiles: no expense, low expense (0.1€–20€), moderate expense (20€–150€) and elevated expense (150€ – maximum). The multinomial regression models make it possible to estimate the relative risks and their confidence intervals by adjusting for the age and sex of the participants.

Multiple linear regression was used to compare pharmaceutical expenditures between immigrants and autochthonous populations, adjusted for the age and gender of the individuals studied. The dependent variable was the natural logarithm of pharmaceutical expenditures during the study period. The logarithm of expense was used because it had a more symmetric distribution than the expense itself. Linear multiple regression models were also obtained to evaluate the differences in the number of containers of medications consumed during the study period.

For the pharmacological study and analysis, medications prescribed to the immigrant population were studied by therapeutic groups. Due to the elevated prescription volume and/or general expense, the nine therapeutic groups selected were the following: *hypertension and cardiac failure*, diabetes related: *insulin *and *oral antidiabetics*, *hyperlipemia*, *asthma and COPD*, *mental health*, *osteoarticular diseases*, *ulcerative diseases *(prophylaxis and treatment), and *infectious diseases*.

Because the immigrant sample included only 24 patients over 64 years of age, all regression models were estimated for patients under 65.

## Results

Table 1 shows the demographic characteristics of the study participants. Women were predominant in the autochthonous group and mean age was greater (48.3 years) with respect to the immigrants (30.7 years). Individuals of Latin American (30%) origin predominated in the immigrant group, followed by individuals from the Maghreb (27%). The percentage of individuals that purchased medications in a six-month period was 53.7% in the immigrant group and 79.2% in the autochthonous group.

**Table 1:** Demographic characteristics of the study populations. Individuals visited at public Primary Care Centers in Lleida, Catalonia (Spain) during 2005.

Variables	Immigrant (n = 1,630)	Spanish-born (n = 4,154)	Total (n = 5,784)
	n (%)	n (%)	n (%)

Sex:			
- Men	795 (48.8)	1,812 (43.6)	2,607 (45.1)
- Women	835 (51.2)	2,342 (56.4)	3,177 (54.9)
Age:			
- Mean (SD)	30.7 (13.9)	48.3 (21.8)	43.4 (21.4)
Country of origin:		4,154 (100%)	4,154 (71.8)
- Latin America	487 (29.9)		487 (8.4)
- Eastern Europe	374 (22.9)		374 (6.5)
- Maghreb	443 (27.1)		443 (7.7)
- Sub-Saharan	298 (18.3)		298 (5.2)
- Other	28 (1.7)		28 (0.5)

There were differences in pharmaceutical expenses between immigrant and autochthonous patients in all age groups and both genders. Table 2 shows the medians for pharmaceutical expenditures, both for the overall sample and for the group of patients that purchased medication in the studied period. Data for immigrants over 64 years of age is not reliable because of the small sample size.

**Table 2:** Total pharmaceutical expenditures (in euros) in the 6 months after a visit to the Primary Care Center.

	**Overall sample**		**Patients that purchased medication**	
	**Immigrants**	**Spanish-born**	**Immigrants**	**Spanish-born**

	**Median (N)**	**Median (N)**	**Median (N)**	**Median (N)**
**Men**				
<= 24	0.0 (206)	5.3 (308)	12.8 (87)	20.2 (183)
25–44	2.3 (492)	6.8 (593)	11.1 (256)	23.0 (379)
45–64	8.9 (93)	49.0 (449)	25.4 (59)	85.9 (363)
>= 65	174.1 (4)	257.7 (462)	174.1 (4)	282.4 (430)

**Women**				
<= 24	13.0 (264)	21.6 (339)	13.3 (126)	16.6 (212)
25–44	4.6 (448)	14.1 (710)	17.1 (264)	29.1 (531)
45–64	15.5 (103)	98.9 (584)	49.9 (73)	122.9 (523)
>= 65	0.0 (20)	286.3 (709)	247.2 (9)	310.5 (670)

When analyzing pharmaceutical expenses by region within the overall immigrant sample, median expenditures were 4.0€ for patients from the Maghreb, about 3.0€ for patients from Latin America and sub-Saharan Africa, and 0€ for patients from Eastern Europe. When the analysis was restricted to patients that purchased medication, median expenditures were greater among patients from the Maghreb (18.3€), followed closely by Latin Americans (17.3€), Eastern Europeans (13.1€), with the lowest expenditures being in the sub-Saharan group (11.0€).

**Figure 1:**
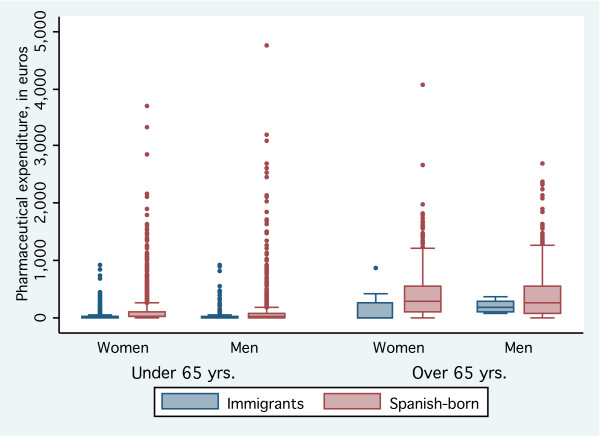
Box-and-whisker plot of pharmaceutical expenditures in 6 months after a primary care visit, by immigrant status.

Figure 1 shows a box-and-whisker plot of the distribution of pharmaceutical expenses, by immigrant and autochthonous patients, over a six-month period. The middle fifty percent of the immigrant group spent between 0€ and 17€, while the middle fifty percent of autochthonous patients spent between 4.1€ and 219.7€. In immigrants, the median expense (3.1€) was less than that of the autochthonous patients (39.6€).

When evaluating the different categories of pharmaceutical expenses (Table 3), it was observed that Spanish-born patients have a greater relative risk of elevated pharmaceutical expenses than immigrants. In men, the relative risk of Spanish-born versus immigrants increases from 1.2 for the low expenditure category to 6.9 for the high expenditure category, with the reference category being not having any pharmaceutical expenditures. In women, the relative risk of Spanish-born versus immigrants increases from 1.6 for the low expenditure category to 5.3 for the high expenditure category.

**Table 3:** Relative risks (RR) of pharmaceutical expenses, by immigrant status, adjusted for age (Multinomial regression). Patients under 65 years old.

**Grup of expenditure**	RR *	**p-value**	**95% CI**
**Men**			
*Low expenditure*			
Spanish-born versus immigrants	1.2	0.12	(0.9, 1.5)
*Moderate expenditure*			
Spanish-born versus immigrants	2.4	<0.001	(1.9, 3.2)
*High expenditure*			
Spanish-born versus immigrants	6.9	<0.001	(4.2, 11.5)
**Women**			
*Low expenditure*			
Spanish-born versus immigrants	1.6	<0.001	(1.3, 1.9)
*Moderate expenditure*			
Spanish-born versus immigrants	2.3	<0.001	(1.8, 2.9)
*High expenditure*			
Spanish-born versus immigrants	5.3	<0.001	(3.5, 8.0)

(*) Relative Risk (RR) refers to the comparison of the categories mentioned with respect to not having any pharmaceutical expenditure. CI: confidence interval.

Note: The category of low expenditure corresponds to the quantity of 0.1€–20€, moderate expenditure is 20€–150€ and high expenditure is 150€ and higher.

We estimated the relative risks of elevated expenses by origin group for both genders together because the sample sizes for each gender were too small. Relative risks of being in the high expenditure category for Spanish-born individuals versus immigrants were 4.6, 95% CI = (2.9, 7.2) for Latin American patients, 19.2, 95% CI = (7.0, 52.6) for Eastern European patients, 4.1, 95% CI = (2.4, 7.0) for patients from the Maghreb, 7.9, 95% CI = (3.2, 19.6) for Sub-Saharan patients and 6.5, 95% CI = (0.8, 52.6) patients from other countries.

Table 4 shows estimates obtained from the linear regression model of the logarithm of medical expenses, by therapeutic group and for both genders. The exponential function of the coefficients corresponds to the ratio between expenses in the Spanish-born group and expenses in the immigrant group. As the table shows, coefficients obtained were positive for almost all therapeutic groups, which indicates greater spending among autochthonous patients, adjusted for sex and age. For both genders, the *mental health *category had the highest ratio of expenses between Spanish-born and immigrant patients. Other therapeutic groups that showed statistically significantly higher expenses in the native-born were *ulcerative diseases*, *infectious diseases *and *osteoarticular diseases *in men and *hypertension *and *ulcerative diseases *in women. A trend towards a higher expenditure for the Spanish-born was seen in the *hyperlipemia *category in men and the *oral anti-diabetics *and *osteoarticular diseases *categories in women.

**Table 4:** Linear regression estimates of the logarithm of the pharmaceutical expenses by immigrant status, adjusted for age. Patients under 65 years old.

**Medication group**	**Coefficient**	Ratio of expenses between Spanish-born and immigrant individuals * exp(coefficient)	**p-value**	**CI (95%)**
**Men**				
Hypertension or cardiac insufficiency	0.17	1.19	0.60	(-0.45, 0.79)
Insulin	-0.11	0.90	0.84	(-1.15, 0.94)
Oral anti-diabetics	0.41	1.51	0.40	(-0.56, 1.38)
Hyperlipemia	0.58	1.79	0.05	(0.01, 1.14)
Asthma or COPD	0.26	1.30	0.43	(-0.39, 0.91)
Mental health	1.29	3.63	<0.001	(0.63, 1.94)
Osteoarticular	0.20	1.22	0.02	(0.03, 0.37)
Ulcerative pathology	0.54	1.72	0.003	(0.19, 0.89)
Infectious pathology	0.33	1.39	0.001	(0.14, 0.52)

**Women**				
Hypertension or cardiac insufficiency	0.52	1.68	0.02	(0.08, 0.95)
Insulin	0.72	2.05	0.20	(-0.40, 1.84)
Oral anti-diabetics	0.88	2.41	0.07	(-0.07, 1.83)
Hyperlipemia	0.20	1.22	0.69	(-0.78, 1.18)
Asthma or COPD	0.34	1.40	0.35	(-0.37, 1.04)
Mental health	0.72	2.05	0.001	(0.28, 1.16)
Osteoarticular	0.19	1.21	0.08	(-0.02, 0.39)
Ulcerative pathology	0.36	1.43	0.01	(0.08, 0.65)
Infectious pathology	0.06	1.06	0.47	(-0.10, 0.22)

* The values in this column indicate the number of times that a Spanish-born individual spends in medication in relation to an immigrant of the same age, sex and medical condition.

We analyzed whether there were differences between the amount of containers of medication purchased at the pharmacy, and we observed that Spanish-born patients purchased more containers than immigrants during the study period. For both genders, the greatest differences were seen in the mental health category, where the Spanish-born purchased, on average, more containers than the immigrants in the studied period (4 containers more in men and 3 more in women). Spanish-born individuals also purchased more containers of insulin and anti-diabetic medications, although the differences did not reach statistical significance.

## Discussion

This work reflects a large difference in pharmaceutical expenses between Spanish-born and immigrant patients. The mean expenditure per immigrant was six times less than that of the Spanish-born group and a larger proportion of the Spanish-born individuals purchased medication (four out of five) with respect to the immigrant group (one out of two). The medication group where the differences were highest was mental health. Relative risks of Spanish-born versus immigrants for being in the highest quartile of pharmaceutical expenditure were 6.9 for men and 5.3 for women. The origin groups that had the lowest risk of being in the high expenditure group were Eastern European and Sub-Saharan patients.

These differences may be influenced by several factors. The first factor is age, since the immigrants treated in primary care centers were younger than their autochthonous counterparts. When the analysis is adjusted for age or the analysis is restricted to individuals under 65 years old, the differences in pharmaceutical expenditures persist, and are statistically significant.

The second factor which may influence pharmaceutical consumption is compliance. In Spain, medications are sold in pre-packaged quantities (e.g. 24 doses), and patients often have to "over-purchase" needed medications to comply with the physicians prescribed treatment, e.g. purchase two packages of 10 daily doses of medication for two weeks treatment, leaving 6 doses unused. The results of the present study show that immigrants purchase less *containers *of medication than autochthonous patients for pharmaceutical treatment within the same medication group. Immigrants are systematically purchasing less medication for treatment of the same types of diseases. This possible lack of adherence to a therapeutic regimen may be based in alternative medical treatments followed by immigrants, communication problems between patients and caregivers, lack of understanding of the Spanish medical system, financial disincentives to "over-purchase" instead of "under medicate", or other factors [13].

The third possible differential factor is a relationship with the price of the medication prescribed. In fact, we have observed that medications prescribed to immigrants are statistically significantly less expensive within the *mental health *category in both genders, as well as the *ulcerative diseases*, *infectious diseases *and *osteoarticular diseases *categories in men and *anti-hypertension *and *ulcerative diseases *categories in women. Given that most patients must pay a part of the cost of pharmaceutical treatment, it is possible that physicians prescribe less expensive medications to immigrants in order to facilitate compliance.

Inequalities in pharmaceutical prescribing by race have been studied intensely in the United States. A study released in the United States in 2005 reports that black patients spend an average of $723.5 less on mental health medications than white patients [14]. Another study, from Briesacher *et al*, found differences in prescribing patterns in patients with diabetes, hypertension and ischemic heart disease [15]. Patients were classified into three groups: non-Hispanic Whites, non-Hispanic Blacks, and Hispanics. In all pathology groups, white patients spent between 20% and 40% more than the rest. Black and Hispanic patients were prescribed less medication and cheaper drugs when treating chronic conditions. These differences remained after adjusting for age, diagnostic pathology, and type of insurance. Gaskin, in a 2006 study in which total pharmaceutical expense was analyzed, estimated that if Black and White individuals had the same demographic characteristics, White individuals would pay 8.9% more in prescription medications than Black individuals [8]. Furthermore, in 2002 Chen analyzed pediatric prescriptions and found that Black children were prescribed less medication than White children, with an odds ratio of 0.67 [16]. As could be expected, children without medical insurance received less medication than those that had insurance. Nevertheless, according to Chen, White children were prescribed more expensive medications than Black, Asian, Hispanic and Native American children, and children from wealthy families spent nearly double the amount on prescription medication as children from families in the lower socioeconomic levels. Surprisingly, the differences between racial and ethnic groups remained when adjusting for socioeconomic and insurance status, even when medical conditions were similar.

Other studies report that immigrants with less economic resources have a decreased probability of emergency, primary care, and pharmaceutical expenses than the naturalized population of the US [17]. Recently arrived immigrants tend to be poorer, and therefore have more economic and cultural barriers to accessing health care due to language and racial or ethnic biases which would also affect their consumption of medications [18,19]. It is important to note that the consumption of medications is related to access to health care services and therefore results observed in countries without a universal health system may be not applicable to countries that have one, such as Spain.

The fourth factor that may affect pharmaceutical consumption is the possibility that immigrants are healthier than native-born patients. There is some evidence of this phenomenon in the literature, which has been described as the "healthy immigrant effect". According to Gushulak, the healthy immigrant effect is related to three conditions, a) existing differences in many diseases between wealthy and less wealthy nations, particularly for lifestyle-associated diseases (diet, exercise, substance abuse); b) differences in immigrants' perception of health and disease (compared to the host population) which causes them to seek and utilize health services at levels less than the host population, and c) the process of migration itself which favors youth and health [20]. Some other factors that can influence the differential health of immigrants are factors related to the host country and its immigration policies. For example, distance to the host country, systematic health screening of immigrants, etc. At present, Spain does not require a health certificate for a residence permit or registration in their municipality of residence, and there are many immigrants that are not identified by normal government oversight.

A study that supports the healthy immigrant effect is Kennedy *et al*, which examined the health outcomes, health behaviors, and socio-economic characteristics of immigrants from a range of countries to the USA, Canada, the UK, and Australia. Some of their findings were: a) the proportion of immigrants with a chronic condition was less prevalent than in the native-born population, b) the foreign-born tended to report better health than the native-born with the exception of Canada, where the native-born reported 'better' health, and c) positive self-selection for recent immigrants was present even for immigrants with relatively low levels of education [21].

Bischoff *et al*, found evidence that the healthy immigrant effect was related to the immigrant's country of origin when studying health disparities between immigrant groups in Switzerland and the Swiss population [22]. Their results show that the self-reported health of "Northern immigrants" (people from Germany and France) did not differ significantly from that of the majority Swiss population, whereas "Southern immigrants" (people from Italy, Former Yugoslavia, Portugal, Spain and Turkey) reported lower levels of health in several areas. Similarly, Wiking *et al *analyzed the association between ethnicity and poor self-reported health in immigrants to Sweden from Poland, Turkey and Iran and the Swedish population [23]. Among men from Iran and Turkey there was a threefold increased risk of poor self-reported health than in Swedes, while the risk was five times higher for women. When socioeconomic status was included in the logistic model the risk decreased slightly. They concluded that the strong association between ethnicity and poor self-reported health seems to be mediated by socioeconomic status, acculturation, and discrimination. In Amsterdam, Reijneveld *et al *found that immigrants from Turkey, Morocco, and former Dutch colonies had increased risks of poor self-reported health compared with the majority population [24]. The authors concluded that the socioeconomic circumstances of ethnic minorities often explain their adverse health status instead of racial and biological factors.

There is only limited data on the health status of immigrants as compared to Spanish-born individuals. Carrasco-Garrido *et al *in 2003 estimated that 80% of Spanish-born individuals and 77% of immigrants assessed their health status as very good or good [25]. In addition, a higher proportion of immigrants reported having made at least one emergency visit in the preceding 12 months, and/or having been hospitalized, in the preceding year. These results would support the hypothesis that immigrants in Spain do not have a better health status than Spanish-born individuals. Consistent with our results, Carrasco-Garrido *et al *also found that less immigrants consumed medications than the Spanish population (42.6% and 49.9%, respectively) [25]. Another study, published in Barcelona (Spain) in 2005, shows clear differences in the use of medications during pregnancy in women from Spain and other Western countries as compared to a group of women composed of women from other areas, such as Africa, Eastern Europe, Asia or South America [26]. Groups formed of Spanish and Western European women were more similar in their pharmaceutical consumption. These two groups took the most folic acid and groups from Asian countries took the least. Immigrant groups in Lleida, when compared to Spanish-born individuals, have poorer living conditions, lower socioeconomic status, and are poorly acculturated due to the recent nature of immigration in Spain. Based on these studies, and the findings mentioned earlier that low socioeconomic level has a strong negative impact on health status, we think that the differences in pharmaceutical consumption that we found in our study are not due to a better health status of the immigrants.

### Limitations

The main limitation of our study is the lack of information on the health status and socioeconomic level of patients, which makes it difficult to determine to what extent observed differences in pharmaceutical consumption are a consequence of existing health differences, socioeconomic disparities or due to compliance or prescribing differences. There is a need for more information on the health status and socioeconomic variables of immigrant groups in Spain, as well as information on both native-born and immigrant groups that use health services.

A second limitation is the insufficient information available by origin group. We have observed differences in pharmaceutical consumption by to zone of origin. Nevertheless, the age-adjusted relative risks estimates are not precise enough to consider our results reliable. It would be interesting to study this issue in future studies.

Among other limitations, we should emphasize the fact that data were not collected on prescriptions written outside the public health care system or for those medications that do not require a prescription. There is a reliable registration system for patients assigned to a family physician and treated in primary care centers [27]. Although about 25% of the Catalan population has some private insurance coverage in addition to NHS coverage, estimations place purchases outside the public health system at 2.2% of all prescriptions sold in the Health Region, which we do not believe is large enough to modify our results substantially [2]. Nevertheless, prescriptions written by the physician do not always reach the pharmacy and incur a pharmaceutical expense. There are some studies that calculate that one in twenty prescriptions are never filled in a pharmacy, with this number being greater in some environments and in patients with specific problems [28]. It would be interesting to quantify this movement in our study area and understand the motivations for this type of behavior.

The findings presented here indicate a need for broader study of this issue, not only from a quantitative perspective but also using a qualitative approach, in order to better understand differential medication expenses between immigrants and native-born individuals [28]. There are some key questions that need to be addressed: Are doctors prescribing less expensive medications to one group as compared to another? Are immigrants not complying with their prescribed treatments? Are immigrants more healthy than the Spanish-born population? Are there important differences between immigrant groups by area of origin?

## Conclusion

Pharmaceutical expenses are much lower for immigrants with respect to Spanish-born patients, when adjusting for age and sex, both in the percentage of prescriptions filled at pharmacies, the number of containers of medication obtained, and the prices of the medications used. Future studies should explore the causes of these differences and whether they produce health inequalities [29]. Furthermore, these studies will be more robust if they are supported by data on the health status and socioeconomic levels of the immigrant and native populations, in order to address the "healthy immigrant effect", as well as to more directly assess factors influencing medication use.

## Conflicts of interests

The author(s) declare that they have no competing interests.

## Authors' contributions

MR participated in the design and the coordination of the study, performed the statistical analysis and helped to draft the manuscript. MCSA, JSG and MCRM participated in the design of the study, interpreted the results and helped to draft the manuscript. AB participated in the statistical analysis and helped to draft the results. LG provided, validated and helped to interpret the pharmaceutical consumption data for the study participants. All authors read and approved the final manuscript.

## Pre-publication history

The pre-publication history for this paper can be accessed here:


